# Exploring the Health Effectiveness of a Physical Activity Program Co-Constructed with Students after the COVID-19 Pandemic

**DOI:** 10.3390/nu15132913

**Published:** 2023-06-27

**Authors:** Aurélie Goncalves, Maxime Deshayes, Béatrice Gisclard, Antony G. Philippe, Caroline Bernal, Sophie Krawczyk, Karim Korchi, Maxence Nogrette, Elodie Charbonnier

**Affiliations:** 1 University of Nîmes, APSY-V, CEDEX 1, F-30021 Nîmes, France; maxime.deshayes@unimes.fr (M.D.); antony.philippe@unimes.fr (A.G.P.); caroline.bernal@unimes.fr (C.B.); elodie.charbonnier@unimes.fr (E.C.); 2 University of Nîmes, PROJEKT, CEDEX 1, F-30021 Nîmes, France; beatrice.gisclard@unimes.fr; 3 La Grande Bobine, 30000 Nîmes, France

**Keywords:** physical activity, health promotion, social design, public involvement, university student

## Abstract

Background: University students have low levels of physical activity and high levels of sedentary behaviors that were exacerbated by the COVID-19 pandemic. Even before the pandemic, there was poor uptake of university sports activities. Therefore, it is essential to develop and test innovative programs to increase students’ motivation to engage in physical activity in order to prevent any future deterioration in their general health. Objective: This exploratory study was conducted to test the effectiveness of a physical activity program that was co-constructed with students. Methods: First, a workshop drawing on social design methodologies and the fundamentals of physical activity programs was conducted to assess students’ needs and desires in terms of physical activity. Second, the effectiveness of a program co-constructed with students on the basis of this workshop was assessed on physical and mental health parameters. The results showed that the workshop outcomes allowed the physical activity program to be tailored to meet students’ expectations (e.g., session duration and type of activities). This innovative physical activity program was found to improve body image, autonomous motivation, and certain physical parameters. At the end of the 8-week program, the adherence rate was 89%, and 83% of the final respondents expressed a wish to re-enroll for the following semester. Conclusions: Involving students and considering their wishes, needs, and objectives could facilitate the development of attractive and innovative programs.

## 1. Introduction

According to the Ottawa Charter, “Health is created and experienced by people in the context of their daily lives: where they learn, work, play and love” [1] (p. 4). Universities should, therefore, be an ideal setting for the implementation of health promotion programs. The Okanagan Charter was based on the notion that university campuses should be “campus cultures of compassion, well-being, equity, and social justice; improve the health of the people who live, learn, work, play and love on our campuses; and strengthen the ecological, social and economic sustainability of our communities and wider society” [2] (p. 2). This is especially important because students are usually in a transitional phase between adolescence and adulthood that prefigures adult behavior [3]. Young adults are exposed to numerous changes that are academic, physical, emotional, and social [4] that can influence key health behaviors, such as alcohol use, physical activity (PA), and diet [3]. It is important to note that entering university is accompanied by a decline in PA [5] and higher levels of sedentary behavior (SB) [6,7], which were even more pronounced during the COVID-19 pandemic [8,9].

The COVID-19 pandemic had a major impact on students’ lifestyles and the way they were taught, especially during periods of lockdown when teachers had to engage in distance learning. During the first lockdown, the closure of universities and the massive use of distance learning led to an increase in SB among students [10,11,12]. One reason for this is the increase in screen time associated with distance learning [10]. Furthermore, active travel, whether to and from campus or to and from classrooms, was considerably reduced, if not halted altogether. It is essential to point out that even before the pandemic, students had been identified as having lifestyles and habits that could be detrimental to their health, including high levels of SB and insufficient PA [6,7]. In other words, the COVID-19 pandemic aggravated pre-existing issues, and this impact is likely to continue, even though the pandemic has waned. In support of this idea, one study found that the COVID-19 pandemic had a negative long-term (8 months) impact on PA and SB [9]. These data point to the need to conduct interventional studies among students aimed at preventing potential deterioration in their health, notably by increasing their PA.

Students, and more generally young people, have a low level of engagement in PA. For example, in a French survey, 25% of young people aged 16–25 years stated that they did not engage in regular PA (less than three times a month or not at all) [13]. This can be explained by the presence of numerous obstacles such as lack of time, workload, or family constraints and poor sociability, meaning that it may be hard to find regular sports partners [13]. Poor uptake of programs aimed at students may be due in part to the fact that most standard PA programs fail to take account of participants’ expectations [14]. As indicated earlier, engagement in PA decreases during the first year at university [15]. Therefore, to overcome this barrier, it is necessary to find new motivations to encourage students’ engagement. As we recover from an unprecedented public health crisis, offering students ways of practicing PA has proved essential, but it is also particularly complex. To overcome these challenges and encourage student engagement, it is essential to develop innovative interventions tailored to students’ specific desires and needs.

Social design appears to be the right way to go in this regard. *Social design* can be defined as “a novel solution to a social problem that is more effective, efficient, sustainable, or just than existing solutions and for which the value created accrues primarily to society as a whole rather than private individuals” [16] (p. 36). Social design focusing on empathy, context, ideation, and iteration appears well suited to address issues of population health. This bottom-up approach has already proven successful in the field of health, where patient participation ensures that interventions targeting lifestyle change are aligned with people’s actual needs [17,18]. *Co-construction* makes it possible to go beyond simple participation. It enhances engagement by making the target audience actors of the required changes [19] and highlights the importance of the work of all stakeholders, including those who are targeted by the program and those who design or implement it. We no longer design *for* others, but *with* and *by* others [20]. UNESCO greatly encouraged this co-construction approach among students during the COVID-19 period [21].

Given the students’ low engagement in university PA programs [22] and the marked deterioration in their lifestyle (notably lower PA and higher SB) during the pandemic, posing a risk to both their physical [23] and mental [24,25] health, the present study tested the effectiveness for the health of a PA program co-constructed with students. In the first two studies, we identified students’ needs and desires in terms of PA in order to create a program that would meet their specific demands, implementing a co-construction approach. In the second study, we assessed the effect of this co-constructed program on students’ health.

## 2. Study 1

### 2.1. Participants

The first challenge was to recruit students in an unfavorable public health context. By the beginning of the study in September 2021, face-to-face teaching had resumed, but with compulsory mask-wearing and social distancing. The practice of PA and sports was therefore complex, with the potential risk of contamination. Nevertheless, all the French Government’s directives were scrupulously followed. It was essential to propose practices that respected social distancing and allowed students to overcome their concerns about resuming PA (sometimes after a long period of inactivity), especially with other students (taking account of the associated fear of contamination).

Students were recruited in person during the induction periods for each course (Humanities, Languages, Psychology, History, Arts and Design, and Law and Sciences), as well as via flyers and social media (i.e., Facebook^®^ and Instagram^®^). The 110 students who agreed to participate in the study were then randomly assigned to either the intervention group or the control group and matched for gender, age, and seniority (55 in each group). Several students subsequently withdrew for various reasons (e.g., time constraints, lack of time, and lack of understanding of the program), some even before the intervention had begun. Therefore, there were 27 students in the intervention group (i.e., participation in the program) and 20 students in the control group (i.e., no workshop and no PA program). Students in the intervention group were invited to take part in a co-construction workshop, and 20 of them agreed to do so (M_age_ = 22.3 ± 3 years).

### 2.2. Design and Co-Construction Process

The main objective of this workshop was to identify the students’ needs and desires. More specifically, we wanted to assess (1) their motivations for engaging in PA and/or sports, (2) the barriers to engaging in PA and/or sports, (3) the types of sports and/or PA that were particularly attractive to them, and (4) their understanding and perception of the communication materials on these topics.

**Figure 1:**
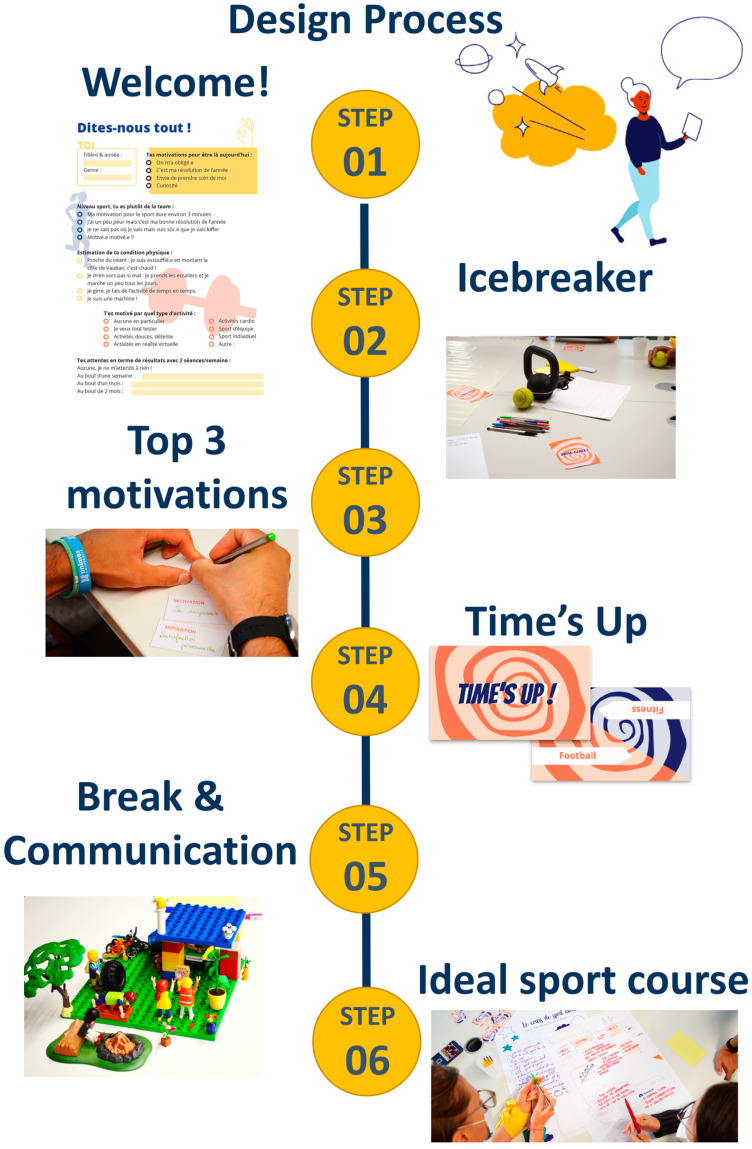
Design co-construction process.

The workshop took place on 22 September 2021 (two half-day sessions). Students could engage in several co-construction activities, divided into three or four groups. Each session lasted approximately 3 h, depending on the context and the number of groups. Each consisted of the following steps: (1) welcome questionnaire (10 min), (2) icebreaker (15 min), (3) motivations (20 min), (4) Time’s Up!© (25 min), (5) break and communication (15–20 min), and (6) ideal course (1.5 h). The step-by-step design process is illustrated in Figure 1.

Step 1. The objective was to get to know each other and set the tone of the workshop. A welcome questionnaire was distributed to each participant. Simplicity and humor were used to frame the workshop in a benevolent and open dialogue. Students were divided into groups according to their curriculum, seniority, and gender to encourage diversity and exchange.

Step 2. The objective of this icebreaker was to share fears, emotions, and feelings, and to encourage participants to listen to each other. One by one, students took cards from a pack and asked questions to their partners. The person asking the questions wrote their answers anonymously on a sheet of paper. All the sheets were collected at the end of the session by the facilitators. These data were used to identify individual barriers and motivations, as well as beliefs and values related to PA.

Step 3. The objective was to identify students’ motivational factors. First, students wrote down their reasons for enrolling in the program and coming to the workshop. Second, they each presented their reasons to the others, thereby making them explicit. A set of pre-established motivations (i.e., gifts, seeing a rapid physical change, feeling proud of oneself, improving one’s health, letting off steam, meeting people, etc.) was combined with the motivations expressed by the participants. The objective was to rank the motivations in a collegial manner rather than according to the type of activity.

Step 4. The objective was to determine how students perceived and understood the different types of PA offered at their university, using the Time’s Up!© (Brussels, Belgium) game format. First, we drew up a list of the sports and physical activities that were potentially available. Players drew a card featuring one of these and then had to make their team guess it by miming or drawing it (even if they did not know what it was!). Once the team had guessed, each member of that team answered three questions printed on a sheet. Question 1: “Are you familiar with this PA? I know what it is/Not at all”. Question 2: “Does this PA tempt you? I am attracted to it/I am afraid of it”. Question 3: “Do you feel capable of doing this PA? I feel capable/Not at all”. This step made us realize that some PAs were not understood by all and that this lack of understanding impeded engagement.

Step 5. During the break, we focused on the communication materials used to encourage people to participate in sports or PA. The project team selected a series of posters featuring a wide variety of slogans, images, and/or photos of PAs and/or sports. The purpose was to interact with the students about the different types of communication used, the various types of messages (i.e., humorous, serious, motivational, and guilt-inducing), and how to avoid misperceived or misunderstood ones. Everyone was free to comment on their feelings and discuss them with other participants.

Step 6. Hales talked about “the power of media design to craft and deploy compelling visions of the future” [26] (p. 2), and students were asked to give free rein to their imagination and have fun developing their ideal course according to their own criteria. This step was divided into three parts (ideation/critique/prototype), and team members could interact not only with each other but also with the other teams. There were no constraints regarding the content of the course, but the students were partly guided by questions about activities, environment, participants, and so on. The teams then exchanged their materials, the objective being to criticize each other’s work and consider all possible reasons for failure. In the last part of the game, the teams had to respond to the criticism by proposing workarounds, and thus devise a realistic roadmap for the course design that suited them. As a *bonus*, students could materially represent their ideal course using Lego© (Billund, Danemark) and Playmobil© (Zirndorf, Germany; see Figure 1). This playful dimension allowed students who were shy or afraid of expressing themselves in front of others to use an alternative means of communication during the oral presentation. After the workshop, a satisfaction questionnaire was sent to all the participants.

### 2.3. Workshop Feedback

These six steps allowed us to identify the students’ desires, motivations, and expectations. Overall, participants considered themselves to have a low level of PA. The welcome questionnaire (Step 1) revealed that most of them (80%) wished to engage in PA in order to take better care of themselves and improve their general health. Step 3 confirmed that their main motivations were health and well-being, followed by social relationships and the desire to surpass oneself. The Time’s Up! game (Step 4) revealed that students were more interested in aerobic activities that ended with flexibility exercises and/or more leisurely activities. They were also interested in exercises that could reduce their back pain. They preferred cardio-boxing and cross-training for fitness training and boxing and were keen to escape the *school* environment. Step 6 allowed us to explore the modalities of the program in greater depth to be able to adapt the sessions and find solutions to the potential inconveniences and causes of dropout identified by participants. Contrary to our expectations, students did not wish to exercise during their lunch break. They also did not want excessively long sessions. Students expressed the desire to be able to exchange personal, even intimate information, and for this reason, they wished to be in a male/female coaching pair, ideally with a student enrolled in the sports science curriculum. They also expressed the wish to have an app in order to communicate with other students between the sessions: a platform they were familiar with and used on a daily basis. Finally, and due to the activities in STEP 4, they specified the activities they would like to practice, which should be different from the activities classically proposed by the university service of PA and sports, and thus break free from an environment considered as “school”. These results allowed us to adapt our PA program to meet the students’ expectations.

### 2.4. Adaptations Made to the Physical Activity Program after the Workshop

Following this workshop, we adapted the PA program (which was due to begin 10 days later) to meet the students’ expectations. We made the following adaptations: (1) modification of the timetable; (2) duration of less than 1.5 h, with 30 min of welcome, exchange, and choice of music, followed by 60 min of PA; (3) supervision of the session by a teacher/student and man/woman pair; (4) creation of a group on the Discord^®^ platform for students to motivate each other to come to the sessions and develop the content of the activities across the weeks; (5) activities such as cross-training and cardio-boxing making up the bulk of the sessions. Once a week, a team sports event was organized (volleyball, baseball 5, tchoukball, spike ball, etc.). All the steps of the program’s conceptualization, from the team’s anticipation to the students’ wishes regarding the adaptations, are summarized in Table 1.

## 3. Study 2

### 3.1. Participants

As previously stated, 110 students who agreed to participate in this study were randomly assigned to one of the two groups, matched for gender, age, and seniority (55 in each group). As several students subsequently withdrew, we assessed 27 students in the intervention group and 20 in the control group at our first measurement timepoint (T0). At our second measurement timepoint (T1), we assessed 24 participants in the experimental group (M_age_ = 22.4 ± 3.3 years) and 17 in the control group (M_age_ = 22.4 ± 7.7 years). Only participants who responded at both T0 and T1 were included in this study. Table 2 presents the participants’ key characteristics.

### 3.2. Materials

Baseline assessments (T0) were carried out in September/October 2021. PA and ST were measured using accelerometers, and physical fitness (body composition) was assessed in person. Psychological variables were collected through an online survey designed using the Qualtrics software (Qualtrics, Provo, UT, USA). The second set of assessments (T1) was conducted in December 2021, nine weeks after the first assessment. At the end of the program, students’ testimonials about the entire intervention were collected on Discord^®^.

#### 3.2.1. Psychological Variables

- **Motivation to engage in physical activity**. We used the French-language *Motivation for PA in a Health Context Scale* [27] to assess participants’ motivations for engaging in PA. The 18 items are rated on a 7-point Likert scale ranging from 1 (*Strongly disagree*) to 7 (*Strongly agree*). This scale measures three dimensions of motivation to engage in PA: autonomous motivation (i.e., includes both the internal and extrinsic motivation of individuals who identify with an activity’s value and how it aligns with their sense of self), controlled motivation (i.e., solely external motivation, where an individual acts out of a desire to obtain external rewards or out of fear of punishment), and amotivation.
- **Body image**. We used the Body Appreciation Scale-2 [28] to measure body image. The 10 items are rated on a 5-point scale ranging from 1 (*Never*) to 5 (*Always*).
- **Anxiety and depressive symptoms**. Anxiety and depressive symptoms were measured using the French-language version of the Hospital Anxiety and Depression Scale [29]. This 14-item scale assesses anxiety (7 items) and depressive symptoms (7 items). The scores range from 0 to 21 for each dimension.
- **Well-being**. Well-being was assessed using the French validation of the Psychological Well-Being Scale [30]. The 18 items are rated on a 6-point Likert scale ranging from 1 (*Disagreement*) to 6 (*Agreement*).

#### 3.2.2. Anthropometric Measures

- **Height** (cm) was measured to the nearest 0.1 cm using a portable stadiometer (Leicester Tanita HR001).
- **Body weight** (kg) was measured using a calibrated scale (Tanita 780 MA-S, Arlington Heights, IL, USA) to the nearest 0.1 kg.
- **Body mass index** (kg/m^2^) was calculated using height and body weight measurements.
- **Body composition (body fat and body muscle)** was assessed using a bioelectrical impedance analysis method with a Tanita 780 MA-S Body, expressed as mass (kg) and percentage (%).

#### 3.2.3. Physical Fitness

- **Flexibility**. Participants completed a traditional sit-and-reach test to measure lower back and hamstring flexibility [30]. In a seated position with the legs extended, students had to reach as far as possible along a measuring line with both hands either on top of each other or side by side. The measurement was repeated three times.
- **Lower limb strength**. To assess muscular strength, participants were seated on a dynamometric chair (LegControl V2.0; Mtraining, Ecole Valentin, France). After a familiarization period consisting of five submaximum isometric contractions, participants performed three 3-s maximum voluntary contractions, separated by 1 min intervals. More specifically, they were requested to contract the knee extensors as hard as they could for 3 s. The maximum value for each participant was used for the statistical analyses.
- **Cardiovascular fitness**. We administered the YMCA 3 min step test [31]. Students had to go up and down 24 steps per minute without stopping. Step frequency was indicated by a metronome set to 96 beats per minute. As soon as they completed the test, students had to sit down. After 5 s, their recovery heart rate was monitored for 1 min, and this heart rate was used to assess their cardiovascular fitness [31,32].
- The method for analyzing and interpreting these variables is described in [33].

#### 3.2.4. Objective Sedentary Time and Physical Activity

PA level and sedentary time (ST) were measured using GT3X tri-axis accelerometers (Actigraph, Pensacola, FL, USA) [34,35]. Participants had to wear the accelerometer on the right side of the hip, adjusted with an elastic belt, day and night for 7 consecutive days. They could only remove it to take a shower or engage in water sports. The Actilife v-6.13.4 Lite Pro software was used to extract the PA and ST values after downloading the data in 10-s epochs. Using the Freedson algorithm, we defined ST as 0–99 counts per minute, and moderate to vigorous PA (MVPA) as >1952 counts per minute [36]. We focused on three variables: the number of sedentary breaks during the day, ST (minutes), and MVPA (minutes). The method for analyzing and interpreting these variables is described in [33].

### 3.3. Intervention Program

In line with the students’ expectations set out in Section 2.4. (*Adaptations made to the physical activity program after the workshop*), the PA program consisted of daily sessions under 1 h in length and was designed to develop strength, endurance, and flexibility/mobility in each session. Activities such as cross-training and cardio-boxing made up most of the program’s sessions, which are described in [33]. A website in WordPress format was created (https://etuzen-sup.unimes.fr/) to describe the steps involved in setting up the intervention.

### 3.4. Statistics

To decide which analyses to perform, we first checked the normality of the data for each variable using the Shapiro-Wilk test. Then, to control for potential differences at T0, we conducted parametric (i.e., Student *t*-test) or nonparametric (i.e., Mann–Whitney test) analyses, according to the normality of the data, to compare means for each dependent variable between the experimental and control groups. Finally, to investigate how each variable changed between T0 and T1 within each group (i.e., experimental and control), we performed parametric (i.e., Student *t*-test) or nonparametric (i.e., Wilcoxon test) analyses.

### 3.5. Results

The means, standard deviations, and comparisons between T0 and T1 for both groups are shown in Table 3. First, the analyses revealed no significant differences between the experimental and control groups at T0 for any of the dependent variables (*p* > 0.05). Second, mean comparisons between T0 and T1 in the experimental group revealed increases in body image (*p* = 0.02, *d* = 0.57), autonomous motivation (*p* = 0.01, *d* = 0.81), flexibility (*p* < 0.01, *d* = 0.77), and the number of breaks (*p* = 0.02, *d* = 0.52). Concerning the other variables, no significant differences emerged between T0 and T1 (*p* > 0.05). In contrast, mean comparisons between T0 and T1 in the control group failed to reveal any significant differences in the dependent variables (*p* > 0.05), except for MVPA, which decreased between T0 and T1 (*p* = 0.03, *d* = 0.63).

Finally, the adherence rate for this program was very high, with 89% of participants completing the program, and 82.6% of respondents at T1 wanting to continue this type of program the following semester. The PA intervention had a very low dropout rate that fell to 5% when we exclusively considered the workshop group. To illustrate the students’ interest in this program, here are some of the comments made by students on Discord^®^:

“*I’ve regained my liking for sport and found real motivation. The sessions were pleasant and tailored to our ability. Although I’m not a great sportswoman, I didn’t feel left out or even overworked. On the contrary, I could be proud of the efforts I made and the outcome of the sessions, especially the wellbeing*”.

(history student)

“*I loved being part of this adventure! I wanted to challenge myself, be part of a project, expose myself to new people and slowly get back into regular PA. This program more than lived up to my expectations! I was able to discover new activities such as cardio-boxing, which I loved!*”.

(psychology student)

## 4. Discussion

Regular PA is essential for lifelong health. However, students have a low level of engagement in PA, which is partly due to the fact that most standard PA programs do not take their expectations into account, and therefore do not meet their needs. Therefore, the main objective of the present study was to promote PA among university students. For this purpose, we conducted two studies. The first one assessed students’ needs and desires in terms of PA in order to create a program that would meet their specific demands. The second one assessed the effects of our co-constructed program on students’ health.

Study 1 highlighted students’ barriers to engaging in PA, such as difficulty fitting the sessions with their timetable, lack of motivation, and unappealing activities. The same barriers to engaging in PA were previously identified among 16- to 25-year-olds [13], and especially among university students [15]. Reasons for early withdrawal are often neglected [37,38]. Our study shed light on these aspects and provided an open-access method for researchers and university staff to replicate the same approach within their universities (https://etuzen-sup.unimes.fr/). 

Study 2 found positive effects of our program on several psychological variables, including body image. This result is in line with a scoping review that highlighted an association between positive body image and greater participation in PA and sports [39]. This effect is all the more interesting, as body appreciation is a mediator of students’ mental health [40,41]. In addition, the results indicated that our program promoted autonomous motivation. A 6-year study analyzing the transition from adolescence to adulthood among 2785 young individuals revealed that elevated autonomous motivation and PA planning were consistently and significantly associated with higher PA [42]. To develop sustained and lifelong PA habits, it is essential to establish positive attitudes toward activity when young, and engaging in sufficient PA leads to better health outcomes [43,44]. These results suggest that our program may promote students’ engagement in PA beyond the program. Consistent with this, at the end of the program, 82.6% of the participants said they wanted to continue this type of PA program the following semester. Regarding anxiety and depressive symptoms, it is important to note that, contrary to our expectations, our program did not seem to have an impact on these parameters, even though many studies highlighted the links between PA and mental health [9,45,46,47,48]. One explanation for this lack of effect could be that the participants in both groups had low baseline anxiety and depressive symptoms. A second explanation may lie in the duration of our program and the frequency of our sessions. Huang and colleagues’ meta-analysis suggested that there should be two or three sessions per week to have a significant effect on anxiety, whereas our program only had one or two sessions per week [49].

Study 2 also yielded interesting results concerning some PA parameters, such as the number of breaks and MVPA. For example, in the control group (i.e., participants who did not participate in the program), the objective level of PA fell between T0 and T1, whereas it was maintained in the experimental group (i.e., participants involved in the PA program). It is important to note that the final measurement was conducted during university exams, which potentially explains the decrease observed in the control group. The absence of a decrease in the experimental group suggests that these participants maintained their PA level despite external constraints (i.e., university exams), revealing a strong investment in the practice of PA and indicating that our intervention had an impact on habits and active living in this group. The present study also revealed the positive effects of our program on flexibility. Activities such as cross-training and cardio-boxing made up most of the program’s sessions. These activities combine both resistance and endurance training at high intensity and are associated with gains in physical fitness (muscle strength and mass, and cardiovascular capacities) [50]. In addition to muscle strength and cardiovascular endurance, flexibility is considered an important health-related fitness measure [51]. In the present study, flexibility increased between T0 and T1 but only in the intervention group, suggesting that the intervention program had beneficial effects on participants’ physical capacity and health. Previous research has shown that flexibility is an important health-related outcome in adults, as it is related to the risk of bone or joint injury, back pain, and difficulty performing activities of daily living [52]. Unsurprisingly, no significant differences were observed concerning the other physical fitness parameters (i.e., muscle strength and cardiovascular fitness). In the literature, most of these beneficial effects were observed after longer PA programs. Numerous studies indicate that high-intensity training programs should contain at least three sessions per week to improve these two parameters [50,53]. In the present study, we did not observe any improvements in lower-limb strength, body muscle percentage, or cardiovascular fitness, suggesting that intensity and duration were too low to induce the physical adaptations observed in a previous study [54]. Taken together, these results on physical fitness (i.e., flexibility) and objective MVPA and ST indicate the efficiency of the intervention program and its positive effects on participants’ health.

Finally, the adherence rate for our program was very high, with 89% of the participants completing the program and 82.6% of the respondents at T1 wanting to continue the following semester. This level of adherence is much higher than that classically observed in the literature. As an illustration, for the adult population, a systematic review of 27 studies reported an adherence rate of 77.5% [55]. The dropout rate was as high as 80% in some studies [38]. We can hypothesize that this discrepancy between our dropout rate and that of *traditional* PA programs is largely explained by the fact that our program was co-constructed with the students. This co-construction method was original in terms of both PA among students and the context of the COVID-19 pandemic. The benefits of involving students have been highlighted in the development of e-health educational interventions to promote PA [56]. Students who participated in the development of the intervention increased their PA, and this was maintained at 2 and 6 months post-intervention. The positive effects of co-construction on commitment can be explained by the fact that it promotes empowerment. Babajanian considers empowerment to be one of the positive externalities of participation [57]. Nevertheless, it should be emphasized that user involvement is not the same as co-design [58]. Arnstein’s seminal study [59] showed that genuine participation implies that the various stakeholders are not simply consulted but have the power to act. In our study, the proposals (e.g., types of activities, days and times of the sessions, coaching pairs, and music playlists) formulated by students were implemented without any distortion or misinterpretation of their words [42].

It is important to note that, as part of an open science approach, the entire procedure for setting up the workshop (Study 1) was made freely available. We very much hope that similar interventions will be implemented in other universities and thus help to increase the number of programs aimed at promoting health and reducing the deterioration in students’ general health. A website in WordPress format was created (https://etuzen-sup.unimes.fr/). One section is dedicated to the co-construction of the PA program, and a brochure about the program contains a detailed description of how to organize the workshop so that other institutions can do so too.

Albeit promising, our results should be interpreted with caution. First, our sample size was relatively small, despite numerous efforts to recruit as many participants as possible. The promotional communication was not very effective and did not allow us to recruit a large number of students. We can hypothesize that students were submerged in all the information given to them at the beginning of the academic year, which was exacerbated by the public health context. Existing infobesity was worsened due to the COVID-19 pandemic [60]. Second, despite the innovative contributions of co-construction and social design, the very originality of this method makes it difficult to generalize the data, as has already been pointed out by Niedderer et al. [61]. How can a local issue be transposed to a larger scale and integrate contextual differences? However, given that this approach seemed to promote participants’ engagement in PA, we believe that it should be prioritized elsewhere. It would have been interesting to compare the effects of a PA program with and without a co-construction workshop. Third, our program was of limited duration and the sessions could have been more frequent. A longer program with more sessions would undoubtedly have increased the effects we observed and allowed additional benefits to be observed, particularly regarding anxiety and depressive symptoms. Nevertheless, it should be noted that we were targeting students who were physically inactive. Therefore, it was necessary to persuade them to adhere to a PA program in order to maintain their commitment over time. In future studies, it would be interesting to compare the effects and adherence levels of a short program like ours with those of a longer program.

## 5. Conclusions

We found that in order to engage students in PA, innovative interventions need to be designed that meet their expectations. This is an essential issue in view of the beneficial effects of PA programs on students’ physical and psychological health. In this sense, running co-construction workshops before offering intervention to students seems to be an interesting avenue to pursue within this specific population.

## Figures and Tables

**Table 1:** Main adaptations to the program after the co-construction workshop.

	Anticipated Features	Students’ Wishes	Adaptations
Schedules	Mainly at lunchtime	Evenings (ideally starting at 6 pm) + Saturday mornings	- Three sessions per week, starting at 6 pm - Saturday morning session starting at 10.30 am—1 session at lunchtime
Duration	1.5 h	1 h	30 min welcome then 60 min session
Supervision	Adapted physical activity teacher	Presence of a woman and presence of students	- Male/female pair - Adapted physical activity teacher + sports science student
Information during the program	At each session	Discord^®^ group session as part of their university timetable	- Discord^®^ moderated by - supervisors - Posting of available sessions on their timetable
Activities	Not determined	Cardio-boxing Cross-training + recreational activities	Everything taken into account

Discord Inc. (San Francisco, CA, USA).

**Table 2:** Participants’ characteristics (*n* = 41).

Characteristics	All Participants *n* (%)	Experimental Group *n* (%)	Control Group *n* (%)
**Sex**			
Female	31 (76)	19 (79.2)	12 (70.6)
Male	10 (24)	5 (20.8)	5 (29.4)
**Level**			
First year	4 (9.8)	0 (0)	4 (23.5)
Second year	13 (31.7)	6 (25)	7 (41.2)
Third year	9 (22)	3 (12.5)	6 (35.3)
Master’s fourth year	11 (26.8)	11 (45.8)	0 (0)
Master’s fifth year	3 (7.3)	3 (12.5)	0 (0)
Undefined	1 (2.4)	1 (4.2)	0 (0)
**Subject**			
Psychology	21 (51.2)	15 (62.5)	6 (35.3)
History/Geography	2 (4.9)	2 (8.3)	0 (0)
Sciences	3 (7.3)	0 (0)	3 (17.6)
Art/Design	8 (19.5)	4 (16.7)	4 (23.5)
Law/Economics/ Management	3 (7.3)	1 (4.2)	2 (11.8)
Literature	1 (2.4)	1 (4.2)	0 (0)
Mathematics	1 (2.4)	0 (0)	1 (5.9)
Others	2 (4.9)	1 (4.2)	1 (5.9)
**Positive COVID-19 test or symptoms**			
Yes	7 (17.1)	3 (12.5)	4 (23.5)
No	30 (73.2)	20 (83.3)	10 (58.8)
Undefined	4 (9.8)	1 (4.2)	3 (17.6)
**Relative with positive COVID-19 test or symptoms**			
Yes	25 (61)	19 (79.2)	6 (35.3)
No	12 (29.2)	4 (16.7)	8 (47.1)
Undefined	4 (9.8)	1 (4.2)	3 (17.6)

**Table 3:** Descriptive analyses of our dependent variables and T0 versus T1 comparison.

	Intervention Group (*M* ± *SD*)			Control Group (*M* ± *SD*)		
	T0	T1	*p* Value	T0	T1	*p* Value
**Psychological variables**						
Anxiety	8.8 (4.0)	8.7 (4.5)	0.96	6.3 (4.8)	6.4 (2.6)	0.36
Depressive symptoms	4.4 (3.1)	4.3 (3.9)	0.87	3.7 (2.8)	4.0 (3.2)	0.34
Well-being	32.1 (4.8)	31.8 (6.2)	0.67	29.8 (4.8)	32 (5.6)	0.29
Body image	31.4 (10.3)	36.0 (10.8)	**0.02**	39 (6.7)	35.5 (7.2)	0.42
Autonomous motivation	40.1 (12.0)	48.5 (11.7)	**0.01**	49.2 (15.9)	44.8 (15.7)	0.85
Controlled motivation	15.4 (5.1)	17.5 (7.1)	0.15	19.4 (5.8)	15.4 (5.9)	0.25
Amotivation	5.1 (3.7)	4.2 (2.1)	0.11	5.2 (3.0)	3.3 (0.5)	0.37
**Anthropometrics**						
Weight (kg)	62.5 (10.5)	62.8 (10.8)	0.35	63.5 (13.0)	64.0 (13.1)	0.12
BMI ^1^ (kg/m^2^)	22.8 (4.0)	22.5 (4.5)	0.49	23.0 (3.8)	23.3 (3.8)	0.15
Body fat (%)	24.7 (7.8)	25.1 (7.8)	0.44	26.6 (8.2)	27.2 (8.1)	0.22
Body muscle (%)	39.7 (5.8)	39.9 (5.6)	0.28	39.8 (6.0)	38.9 (7.8)	0.49
**Physical fitness**						
Flexibility (cm)	1.4 (9.7)	−4.3 (10.4)	**0.001**	4.4 (9.1)	4.0 (12.7)	0.83
Lower limb strength (N)	410.6 (151.6)	415.7 (156.5)	0.66	400.7 (110.3)	422.5 (154.0)	0.37
Cardiovascular fitness ^2^	105.5 (23.2)	102.4 (20.0)	0.19	104.8 (21.3)	101.8 (20.8)	0.32
**Objective ST ^3^ and PA ^4^ (min/day)**						
ST	805.1 (61.1)	816.2 (47.1)	0.38	824.7 (29.7)	831.1 (55.1)	0.61
Number of breaks	19.4 (3.0)	21.3 (3.0)	**0.02**	20.9 (3.1)	22.2 (3.2)	0.12
MVPA ^5^	52.0 (17.4)	48.2 (17.4)	0.28	50.4 (18.9)	39.6 (21.3)	**0.034**

^1^ BMI: body mass index; ^2^ Cardiovascular fitness measured with YMCA HR3 test; ^3^ ST: sedentary time sedentary time; ^4^ PA: Physical activity; ^5^ MVPA: moderate to vigorous physical activity.

## Data Availability

Data available on request due to restrictions eg privacy or ethical. The data presented in this study are available on request from the corresponding author.

## References

[1] World Health Organization (1986). Ottawa Charter for Health Promotion.

[2] International Conference on Health Promoting Universities & Colleges (2015). Okanagan Charter: An International Charter for Health Promoting Universities & Colleges.

[3] Nelson M. (2009). Alcohol Use, Eating Patterns, and Weight Behaviors in a University Population. Am. J. Health Behav..

[4] Bray S.R., Born H.A. (2004). Transition to University and Vigorous Physical Activity: Implications for Health and Psychological Well-Being. J. Am. Coll. Health.

[5] Kwan M.Y., Cairney J., Faulkner G.E., Pullenayegum E.E. (2012). Physical Activity and Other Health-Risk Behaviors During the Transition Into Early Adulthood: A Longitudinal Cohort Study. Am. J. Prev. Med..

[6] Castro O., Bennie J., Vergeer I., Bosselut G., Biddle S.J.H. (2020). How Sedentary Are University Students? A Systematic Review and Meta-Analysis. Prev. Sci..

[7] Castro O., Bennie J., Vergeer I., Bosselut G., Biddle S.J.H. (2018). Correlates of Sedentary Behaviour in University Students: A Systematic Review. Prev. Med..

[8] Wilson O.W.A., Holland K.E., Elliott L.D., Duffey M., Bopp M. (2021). The Impact of the COVID-19 Pandemic on US College Students’ Physical Activity and Mental Health. J. Phys. Act. Health.

[9] Goncalves A., Le Vigouroux S., Charbonnier E. (2021). University Students’ Lifestyle Behaviors during the Covid-19 Pandemic: A Four-Wave Longitudinal Survey. Int. J. Environ. Res. Public Health.

[10] Gallè F., Sabella E.A., Ferracuti S., De Giglio O., Caggiano G., Protano C., Valeriani F., Parisi E.A., Valerio G., Liguori G. (2020). Sedentary Behaviors and Physical Activity of Italian Undergraduate Students during Lockdown at the Time of COVID−19 Pandemic. Int. J. Environ. Res. Public Health.

[11] Gallo L.A., Gallo T.F., Young S.L., Moritz K.M., Akison L.K. (2020). The Impact of Isolation Measures Due to COVID-19 on Energy Intake and Physical Activity Levels in Australian University Students. Nutrients.

[12] Rodríguez-Larrad A., Mañas A., Labayen I., González-Gross M., Espin A., Aznar S., Serrano-Sánchez J.A., Vera-Garcia F.J., González-Lamuño D., Ara I. (2021). Impact of COVID-19 Confinement on Physical Activity and Sedentary Behaviour in Spanish University Students: Role of Gender. Int. J. Environ. Res. Public Health.

[13] Muller J. (2022). Les Jeunes Éloignés Du Sport n’y Sont Pas Hostiles Mais Sont Freinés Par Trop de Contraintes. Crédoc Consomm. Modes Vie.

[14] Perignon M., Dubois C., Gazan R., Maillot M., Muller L., Ruffieux B., Gaigi H., Darmon N. (2017). Co-Construction and Evaluation of a Prevention Program for Improving the Nutritional Quality of Food Purchases at No Additional Cost in a Socioeconomically Disadvantaged Population. Curr. Dev. Nutr..

[15] Thomas A.M., Beaudry K.M., Gammage K.L., Klentrou P., Josse A.R. (2019). Physical Activity, Sport Participation, and Perceived Barriers to Engagement in First-Year Canadian University Students. J. Phys. Act. Health.

[16] Phills J., Deiglmeier K., Miller D. (2008). Rediscovering Social Innovation. Stanf. Soc. Innov. Rev..

[17] Rodgers M.M., Cohen Z.A., Joseph L., Rossi W. (2012). Workshop on Personal Motion Technologies for Healthy Independent Living: Executive Summary. Arch. Phys. Med. Rehabil..

[18] Van Hecke A., Verhaeghe S., Grypdonck M., Beele H., Flour M., Defloor T. (2011). Systematic Development and Validation of a Nursing Intervention: The Case of Lifestyle Adherence Promotion in Patients with Leg Ulcers. J. Adv. Nurs..

[19] Speake H. (2018). Exploring the User-Centred Design of a Physical Activity Pathway in NHS Care. Ph.D. Thesis.

[20] Anselma M., Altenburg T.M., Emke H., van Nassau F., Jurg M., Ruiter R.A.C., Jurkowski J.M., Chinapaw M.J.M. (2019). Co-Designing Obesity Prevention Interventions Together with Children: Intervention Mapping Meets Youth-Led Participatory Action Research. Int. J. Behav. Nutr. Phys. Act..

[21] UNESCO COVID-19: Reopening and Reimagining Universities, Survey on Higher Education through the UNESCO National Commissions. https://unesdoc.unesco.org/ark:/48223/pf0000378174.

[22] ONAPS, ANESTAPS (2022). Enquête sur la Pratique d’Activités Physiques et Sportives et La Sédentarité à l’Université.

[23] Pengpid S., Peltzer K. (2019). Sedentary Behaviour, Physical Activity and Life Satisfaction, Happiness and Perceived Health Status in University Students from 24 Countries. Int. J. Environ. Res. Public Health.

[24] Lee E., Kim Y. (2019). Effect of University Students’ Sedentary Behavior on Stress, Anxiety, and Depression. Perspect. Psychiatr. Care.

[25] Aceijas C., Waldhäusl S., Lambert N., Cassar S., Bello-Corassa R. (2017). Determinants of Health-Related Lifestyles among University Students. Perspect. Public Health.

[26] Hales D. (2013). Design Fictions an Introduction and Provisional Taxonomy. Digit. Creat..

[27] Boiché J., Gourlan M., Trouilloud D., Sarrazin P. (2019). Development and Validation of the ‘Echelle de Motivation Envers l’Activité Physique En Contexte de Santé’: A Motivation Scale towards Health-Oriented Physical Activity in French. J. Health Psychol..

[28] Kertechian S., Swami V. (2017). An Examination of the Factor Structure and Sex Invariance of a French Translation of the Body Appreciation Scale-2 in University Students. Body Image.

[29] Lepine J.P., Godchau M., Brun P. (1985). Anxiety and Depression in Inpatients. Lancet.

[30] Wells K.F., Dillon E.K. (1952). The Sit and Reach—A Test of Back and Leg Flexibility. Res. Q. Am. Assoc. Health Phys. Educ..

[31] Kasch F.W. (1961). A Comparison of Exercise Tolerance of Postrheumatic and Normal Boys. J. Assoc. Phys. Ment. Rehabil..

[32] Santo A.S., Golding L.A. (2003). Predicting Maximum Oxygen Uptake from a Modified 3-Minute Step Test. Res. Q. Exerc. Sport.

[33] Goncalves A., Bernal C., Korchi K., Nogrette M., Deshayes M., Philippe A.G., Gisclard B., Charbonnier E. (2022). Promoting Physical Activity Among University Students During the COVID-19 Pandemic: Protocol for a Randomized Controlled Trial. JMIR Res. Protoc..

[34] Cattelier D.J., Hannan P.J., Murray D.M., Aaddy C.L., Conway T.L., Yang S., Rice J.C. (2005). Imputation of Missing Data When Measuring Physical Activity by Accelerometry. Med. Sci. Sports Exerc..

[35] Trost S.G., McIver K.L., Pate R.R. (2005). Conducting Accelerometer-Based Activity Assessments in Field-Based Research. Med. Sci. Sports Exerc..

[36] Freedson P.S., Melanson E., Sirard J. (1998). Calibration of the Computer Science and Applications, Inc. Accelerometer. Med. Sci. Sports Exerc..

[37] Williams N.H., Hendry M., France B., Lewis R., Wilkinson C. (2007). Effectiveness of Exercise-Referral Schemes to Promote Physical Activity in Adults: Systematic Review. Br. J. Gen. Pract..

[38] Gidlow C., Johnston L.H., Crone D., James D. (2005). Attendance of Exercise Referral Schemes in the UK: A Systematic Review. Health Educ. J..

[39] Sabiston C.M., Pila E., Vani M., Thogersen-Ntoumani C. (2019). Body Image, Physical Activity, and Sport: A Scoping Review. Psychol. Sport Exerc..

[40] Han B., Du G., Yang Y., Chen J., Sun G. (2023). Relationships between Physical Activity, Body Image, BMI, Depression and Anxiety in Chinese College Students during the COVID-19 Pandemic. BMC Public Health.

[41] Ryff C.D. (1989). Happiness is everything, or is it? Explorations on the meaning of psychological well-being. J. Pers. Soc. Psychol..

[42] Courtney J.B., Li K., Nelson T.L., Nuss K.J., Haynie D.L., Iannotti R.J., Simons-Morton B.G. (2021). Autonomous Motivation and Action Planning Are Longitudinally Associated with Physical Activity during Adolescence and Early Adulthood. Psychol. Sport Exerc..

[43] Ridgers N.D. (2012). Youth Physical Activity and Sedentary Time and Associations with Cardiometabolic Health. Evid.-Based Nurs..

[44] Ekelund U., Luan J., Sherar L.B., Esliger D.W., Griew P., Cooper A. (2012). Moderate to Vigorous Physical Activity and Sedentary Time and Cardiometabolic Risk Factors in Children and Adolescents. JAMA.

[45] Talapko J., Perić I., Vulić P., Pustijanac E., Jukić M.J., Bekić S., Meštrović T.M., Škrlec I. (2021). Mental Health and Physical Activity in Health-Related University Students during the COVID-19 Pandemic. Healthcare.

[46] Charbonnier E., Le Vigouroux S., Goncalves A. (2021). Psychological Vulnerability of French University Students during the COVID-19 Pandemic: A Four-Wave Longitudinal Survey. Int. J. Environ. Res. Public Health.

[47] Luo Q., Zhang P., Liu Y., Ma X., Jennings G. (2022). Intervention of Physical Activity for University Students with Anxiety and Depression during the COVID-19 Pandemic Prevention and Control Period: A Systematic Review and Meta-Analysis. Int. J. Environ. Res. Public Health.

[48] Herbert C. (2022). Enhancing Mental Health, Well-Being and Active Lifestyles of University Students by Means of Physical Activity and Exercise Research Programs. Front. Public Health.

[49] Huang X., Wang Y., Zhang H. (2023). Effects of Physical Exercise Intervention on Depressive and Anxious Moods of College Students: A Meta-Analysis of Randomized Controlled Trials. Asian J. Sport Exerc. Psychol..

[50] MacInnis M.J., Gibala M.J. (2017). Physiological Adaptations to Interval Training and the Role of Exercise Intensity. J. Physiol..

[51] Caspersen C.J., Powell K.E., Christenson G.M. (1985). Physical Activity, Exercise, and Physical Fitness: Definitions and Distinctions for Health-Related Research. Public Health Rep..

[52] Garber C.E., Blissmer B., Deschenes M.R., Franklin B.A., Lamonte M.J., Lee I.-M., Nieman D.C., Swain D.P. (2011). Quantity and Quality of Exercise for Developing and Maintaining Cardiorespiratory, Musculoskeletal, and Neuromotor Fitness in Apparently Healthy Adults. Med. Sci. Sports Exerc..

[53] Lopez P., Taaffe D.R., Newton R.U., Galvão D.A. (2021). Resistance Exercise Dosage in Men with Prostate Cancer: Systematic Review, Meta-Analysis, and Meta-Regression. Med. Sci. Sports Exerc..

[54] Philippe A.G., Goncalves A., Martinez C., Deshayes M., Charbonnier E. (2022). Can an Eight-Session Multicomponent Physical Exercise Program Reduce Fall Risk and Fear of Falling among the Elderly?. Int. J. Environ. Res. Public Health.

[55] Albert F.A., Crowe M.J., Malau-Aduli A.E.O., Malau-Aduli B.S. (2020). Functionality of Physical Activity Referral Schemes (PARS): A Systematic Review. Front. Public Health.

[56] Sabooteh S., Feizi A., Shekarchizadeh P., Shahnazi H., Mostafavi F. (2021). Designing and Evaluation of E-Health Educational Intervention on Students’ Physical Activity: An Application of Pender’s Health Promotion Model. BMC Public Health.

[57] Babajanian B. (2015). Promoting Empowerment? The World Bank’s Village Investment Project in Kyrgyzstan. Cent. Asian Surv..

[58] Sánchez de la Guía L., Puyuelo Cazorla M., De-Miguel-Molina B. (2017). Terms and Meanings of “Participation” in Product Design: From “User Involvement” to “Co-Design”. Des. J..

[59] Arnstein S.R. (1969). A Ladder of Citizen Participation. J. Am. Inst. Plan..

[60] Hong H., Kim H.J. (2020). Antecedents and Consequences of Information Overload in the COVID-19 Pandemic. Int. J. Environ. Res. Public Health.

[61] Niedderer K., Ludden G., Clune S.J., Lockton D., Mackrill J., Morris A., Cain R., Gardiner E., Evans M., Gutteridge R. (2016). Design for Behaviour Change as a Driver for Sustainable Innovation: Challenges and Opportunities for Implementation in the Private and Public Sectors. Int. Des. J..

